# Protocol of the STRess at Work (STRAW) Project: How to Disentangle Day-to-Day Occupational Stress among Academics Based on EMA, Physiological Data, and Smartphone Sensor and Usage Data

**DOI:** 10.3390/ijerph17238835

**Published:** 2020-11-27

**Authors:** Larissa Bolliger, Junoš Lukan, Mitja Luštrek, Dirk De Bacquer, Els Clays

**Affiliations:** 1 Department of Public Health and Primary Care, Ghent University, C. Heymanslaan 10, 9000 Ghent, Belgium; dirk.debacquer@ugent.be (D.D.B.); els.clays@ugent.be (E.C.); 2 Department of Intelligent Systems, Jožef Stefan Institute, Jožef Stefan International Postgraduate School, Jamova Cesta 39, 1000 Ljubljana, Slovenia; junos.lukan@ijs.si (J.L.); mitja.lustrek@ijs.si (M.L.)

**Keywords:** ecological momentary assessment (EMA), physiological data, smartphone sensor and usage data, day-to-day occupational stress, academic settings

## Abstract

Several studies have reported on increasing psychosocial stress in academia due to work environment risk factors like job insecurity, work-family conflict, research grant applications, and high workload. The STRAW project adds novel aspects to occupational stress research among academic staff by measuring day-to-day stress in their real-world work environments over 15 working days. Work environment risk factors, stress outcomes, health-related behaviors, and work activities were measured repeatedly via an ecological momentary assessment (EMA), specially developed for this project. These results were combined with continuously tracked physiological stress responses using wearable devices and smartphone sensor and usage data. These data provide information on workplace context using our self-developed Android smartphone app. The data were analyzed using two approaches: 1) multilevel statistical modelling for repeated data to analyze relations between work environment risk factors and stress outcomes on a within- and between-person level, based on EMA results and a baseline screening, and 2) machine-learning focusing on building prediction models to develop and evaluate acute stress detection models, based on physiological data and smartphone sensor and usage data. Linking these data collection and analysis approaches enabled us to disentangle and model sources, outcomes, and contexts of occupational stress in academia.

## 1. Introduction

While work in academia used to be seen as relatively stress free, the number of studies reporting increasing psychosocial stress in the field of university research is growing. Job insecurity, lack of personal or professional development at work, incongruence between the researcher and the institute concerning freedom and independence at work, and lack of recognition of peers, are factors associated with an increased level of stress among researchers [1]. A study on burnout among academics reported work-family conflict, being involved in earning research grants, administrative paperwork, and overall high quantitative workload as further work environment risk factors experienced by researchers, making it an interesting target group for occupational stress research [2].

Over the past decades, one focus of researchers in the field of psychosocial occupational epidemiology has been set on various chronic exposures to psychosocial stress and its adverse impact on chronic disease outcomes [3,4]. The influence on mental and cardiovascular health in particular has received considerable attention [5,6]. The job-demand-control-support model [7] and the effort-reward-imbalance model [8] are two leading frameworks in the field of stress research, recognized to model psychosocial work environments. Traditional research focusing on chronic stress experiences represents a generalization of working life reality. However, dynamic patterns of stress perception like short-term episodes of stress at work inducing acute physiological stress responses, are not accounted for.

The STRess At Work (STRAW) project is based on a collaboration between a research team of the Department of Public Health and Primary Care at Ghent University, Belgium and a research team of the Department of Intelligent Systems at the Jožef Stefan Institute, Slovenia. The experience and expertise of these two fields present the opportunity to collaborate on an innovative combination of several aspects: (1) we focus on day-to-day stress and not on chronic stress, (2) we detect stress in real-world settings, meaning at work and not in lab studies in which participants get exposed to artificially created stress situations, and (3) we measure work environment risk factors and stress outcomes repeatedly, meaning more than twice, over a short period of time as compared to traditional longitudinal and follow-up studies. Additionally, physiological responses to stress and smartphone sensor and usage data are measured continuously.

The work environment risk factors and stress outcomes are measured via an ecological momentary assessment (EMA). This is a research method which allows participants to report on their experiences in real-time and in real-world settings. Data are collected repeatedly over a certain period (often several days) and, more recently, through digital platforms like smartphone applications [9]. Several recent studies have shown the feasibility of using an EMA approach to investigate work stress experiences [10,11,12]. There are three main benefits to it compared to traditional epidemiological methods. Firstly, on top of having data on between-person variations, within-person variations in day-to-day experiences are taken into account. Secondly, in the STRAW project, the EMA results, as repeated data, were analyzed in combination with the baseline data collected in an initial online survey. This offers the opportunity to explore relations between chronic and day-to-day stress experiences, while the latter captures fluctuations in work experiences, which increases the understanding of psychosocial stress experiences [13]. Thirdly, retrospective recall bias can be limited, since the data are collected in real-time, possibly during or shortly after an event or situation of interest occurred [14,15].

Physiological responses to stress are measured with a wristband. While psychosocial work stress and its operating pathophysiological mechanisms are a complex phenomenon [16], the physiological nature of acute stress in humans has been well documented [17]. Exposure to a stress stimulus induces physiological activation of the sympathetic nervous system, followed by a restoration phase through the activation of the parasympathetic nervous system. This process has been monitored via physiological signals such as heart rate and blood pressure within controlled lab experiments, where the participant gets exposed to an artificially created stress situation such as solving a mathematical equation [17,18]. Compared to these studies, the STRAW project collected data on stress experiences in real-world work environments. Through machine-learning, a computer science method which focuses on building prediction models from previously collected data, acute stress detection models can be developed and evaluated. Several studies reported success when it came to differentiating between acute stress conditions and periods without any stress experiences, by combining different physiological signals, mainly heart rate variability and electrodermal activity, which were measured in the STRAW project [19,20,21]. Furthermore, physical activity and stress can cause similar physiological responses. To be able to distinguish these two, accelerometer data were collected. Physical activity is also relevant because it represents a context which might affect stress experiences. An activity-recognition method has previously been developed, which recognizes participants’ activities from accelerometer data [22].

This paper describes the protocol of a study in which we used a novel combination of methodological approaches to explore day-to-day stress in real-world settings among academic personnel. The study aims to answer the following main research question: How to best model relations between (1) work environment risk factors, (2) stress outcomes experienced in occupational settings, (3) physiological stress parameters, and (4) context as inferred from smartphone sensor and usage data in office-based workers employed in academic settings?

## 2. Materials and Methods

### 2.1. Study Design and Study Population

The STRAW project combines an electronic daily diary study in form of an ecological momentary assessment (EMA) with physiological data, and smartphone sensor and usage data monitoring.

The population of interest was healthy adults with a sedentary office-based job, employed in an academic setting while their educational level was of no importance. Further inclusion criteria were that participants needed to use an Android smartphone, work at least 80 % of the full-time workweek (increased exposure to work environment risk factors was required), agree to install the app on their personal smartphone or the smartphone they use most during office hours and for work-related purposes, agree to wear the Empatica wristband continuously during waking hours of working days, and have permission from their superiors to participate in data collection during working hours.

The stress detection model previously developed, using the Empatica wristband, was constructed from lab recordings of 21 participants and real-world recordings of 5 participants [21]. In the STRAW project, a higher sample was required to analyze the relation to work environment risk factors and stress outcomes, and due to the broader and more complex study protocol. We aimed to include at minimum 50 participants (approximately 25 in each country) after drop-out. Having participated on 10 out of 15 days was considered full participation. Participants who dropped out prematurely (i.e., after 9 days or less) were be replaced by new participants.

Since this was a prospective observational study, no health-related risks or benefits for participants were expected. The STRAW project received ethical clearance from the Commission of Medical Ethics of the Ghent University Hospital, Belgium (No. EC/2019/1091) and the Ethics Committee of the Faculty of Arts at the University of Ljubljana, Slovenia (No. 168-2019).

### 2.2. Procedure and Data Collection Methods

Potential participants were recruited via convenience sampling, contacted via face-to-face interaction or email, and informed about the STRAW project with a structured information letter. Further recruitment strategies included reaching out to the personal network of the researchers, printed flyers, and posts on internal communication pages. Two academic institutions in Belgium and two academic institutions in Slovenia were contacted. Before recruitment in an institution started, the head of the department or research group was contacted to receive approval to contact their employees directly. Interested persons were screened for eligibility with our inclusion criteria; they were then included, and more detailed information about the data collection process was shared with the participants via email and our STRAW website (https://strawproject.eu/).

During a briefing at the participant’s office, participants signed a printed informed consent form, were guided through the installation and usage of the STRAW app on their smartphone and the usage of the Empatica wristband, had their blood pressure and heart rate measured, and were asked to wear the Empatica wristband during the first night to collect physiological baseline data during their sleep. The blood pressure and heart rate are measured twice per session with the clinically certified Omron M6 in Belgium and the Omron M10-IT in Slovenia. Furthermore, they received access to an information document which summarized all relevant information given during the briefing, including contact details to reach the researchers in case of questions or problems.

**Figure 1:**
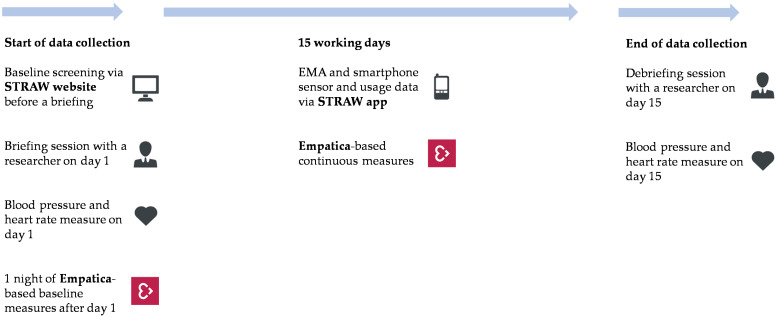
Data collection procedure.

During a debriefing, participants had their blood pressure and heart rate measured again. Participants in Belgium received a 30 EUR voucher as a monetary reimbursement for their efforts. For the partner institute in Slovenia, as a public institution, providing incentives to study participants is legally very difficult, so no such monetary incentives were given to participants in Slovenia. This may limit the comparability of both samples, but since it was a modest monetary reimbursement, the impact is expected to be limited. Moreover, all participants received a personalized feedback report based on their own study results at the end of their participation as an incentive. An illustrated description of the data collection procedure can be found in Figure 1.

Once participants were included, they were asked to complete an online survey on LimeSurvey, accessible via our STRAW website. The survey consisted of an electronic informed consent form, items concerning demographics, work- and health-related information, and questionnaires which were greatly overlapping with the questionnaires included in the EMA (see Table A1 for an overview). These results served as baseline data.

Participants were then asked to answer the EMAs and to wear the Empatica wristband for 15 consecutive working days (the briefing marks the first and the debriefing the last day of data collection). The smartphone sensor and usage data were collected during the same period. While the EMAs asked for active participation, physiological data and smartphone sensor and usage data were collected automatically (given that the participants were wearing the Empatica wristband as planned). Data were only collected during weekdays to decrease participant burden. EMAs and smartphone sensor and usage data automatically stopped at Friday around midnight and picked up on Monday after midnight again. Participants did not need to wear the Empatica wristband during weekends.

**Figure 2:**
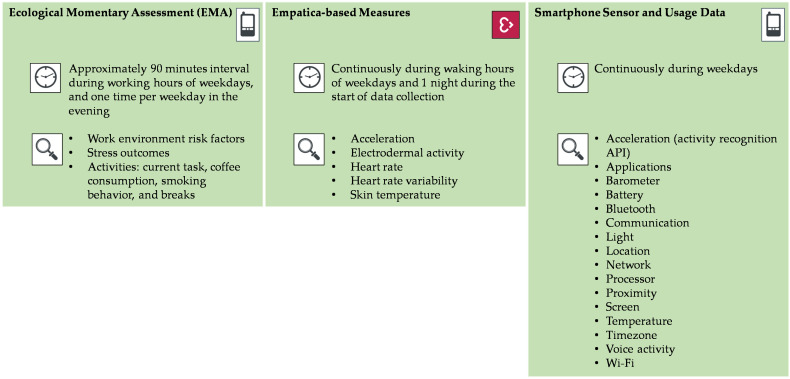
Three parts of data collection methods.

The data collection period consisted of three parts: (1) the EMA via the STRAW app, (2) the physiological data measured with Empatica wristbands, and (3) smartphone sensor and usage data via the STRAW app. An overview of the data collection parts can be found in Figure 2.

#### 2.2.1. Ecological Momentary Assessment (EMA)

##### EMA Content Development and Description

The content of the EMA was specifically designed for the STRAW project, based on a several step process. Firstly, two pre-studies were conducted for initial content ideas and potentially suitable questionnaires: (1) a systematic literature review focusing on work environment risk factors causing day-to-day occupational stress using repeated measurements (registered on PROSPERO (https://www.crd.york.ac.uk/prospero/), ID: CRD42018105355), and (2) a focus group study investigating causes for occupational stress among academic and non-academic office-based workers. Publications on both pre-studies are in preparation. Secondly, the final set of questionnaires was then chosen due to their relevance in the field of psychosocial occupational epidemiology, reliability and validity, and based on two frameworks: (1) the 6th European working conditions survey [23] for the work environment risk factors and (2) the stress process model [24] for the stress outcomes. Thirdly, the complete EMA protocol, especially the questionnaires, were extensively tested within the research team, among volunteering colleagues, and five participants of a pilot study which took place at Ghent University from February until March 2020.

**Figure 3:**
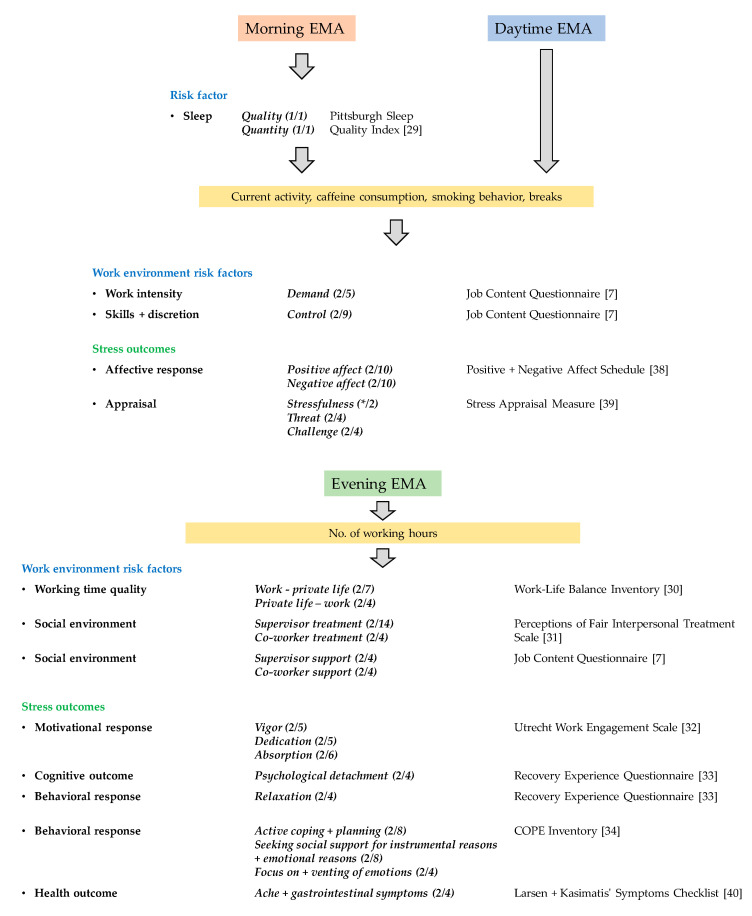
A simplified overview of one day of ecological momentary assessments (EMAs). Column description from left to right: work environment risk factors/stress outcomes, questionnaire subscales, and questionnaires used in EMA. Numbers in brackets: number of items per EMA/total number of items in the questionnaire subscale.

The items included in the STRAW project were chosen based on their factor loadings, reliability, content, applicability to be asked repeatedly, and their previous use in comparable studies. The content and structure per EMA type can be found in Figure 3. See Table A1 for an overview of the included questionnaires.

**Figure 4:**
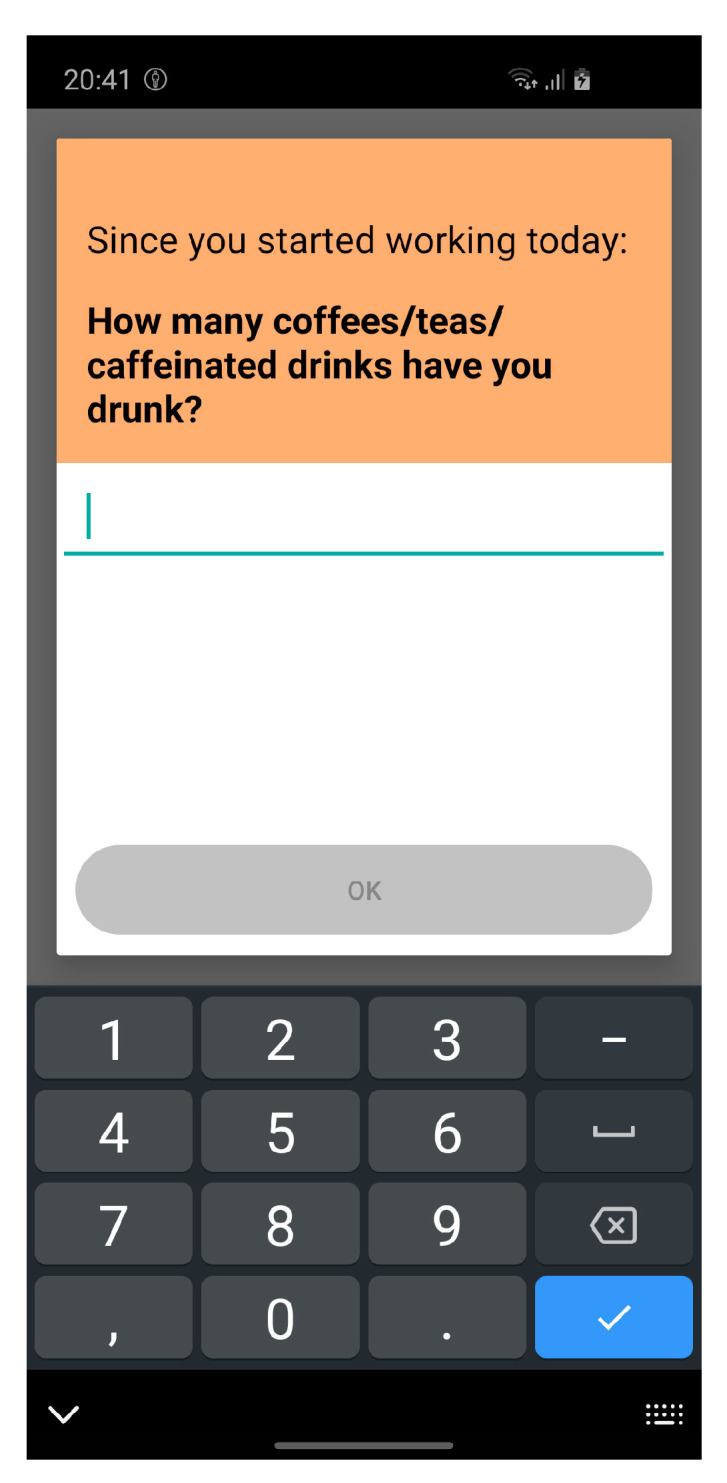
Example of an EMA item. An EMA is divided into three parts: (1) instruction, emphasizing the time frame about which the item is asking, (2) item (question or statement), and (3) participants’ answer.

Response options were based on the original questionnaires and adapted to make them suitable to be answered several times per day. These response options are mostly Likert scales (e.g., “Since the last questionnaire: my job allowed me to make a lot of decisions on my own”, answered on a 4-point Likert scale ranging from “I strongly disagree” (0) to “I strongly agree” (3)) and binary answer scales (yes/no). The work activities were selected from a pre-defined list, and only a few questions were answered by using numbers in open-response format (example shown in Figure 4).

When no official questionnaire translations in Dutch and Slovenian were publically available, the content was translated from English to Dutch and Slovenian by native speakers and official translators, using the back-translation technique.

Our STRAW app was developed using the AWARE framework [25] as a starting point, but the EMA capabilities were significantly upgraded. This app implemented the EMAs and presented them as a well-structured and user-friendly electronic diary on the participant’s own smartphone to maximize participant adherence.

The EMA protocol included three different types: (1) morning EMA, (2) daytime EMA, and (3) evening EMA. Since the EMAs were answered with a relatively high frequency, a few items per questionnaire subscale focusing on work environment risk factors and stress outcomes as our main interest were automatically and randomly selected per EMA. For example: to measure the work environment risk factor “social environment”, two out of four items of the questionnaire subscale “supervisor support” of the job content questionnaire were answered by the participant. One special case is the questionnaire subscale “stressfulness”. The participants were asked if they experienced a particular event that created tension in them. In case of yes, they received further sub-questions from the questionnaire subscales “threat” and “challenge”. In case of no, they were asked if the overall period since the last questionnaire created tension in them and they were asked about the “stressfulness” of such period.

**Figure 5:**
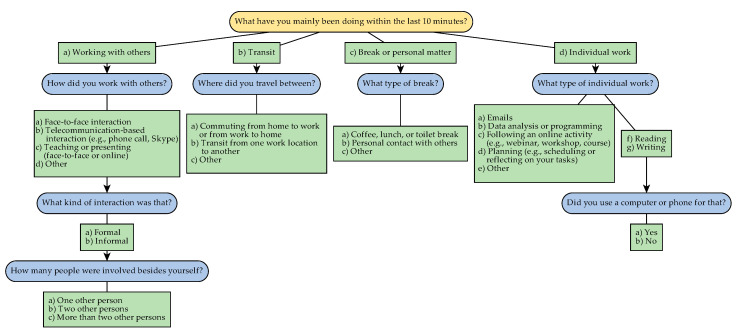
Overview of activities during working hours, included in the EMA.

In addition to the work environment risk factors and stress outcomes, health-related behaviors, which are well known to have an impact on physiological stress responses, such as caffeine consumption, smoking, and breaks from work (especially when breaks include physical activity), were included in the EMA. Questions about health-related behaviors were personalized during the briefing, for instance non-smoking participants were not asked about smoking behavior. Additionally, the participants registered their total working hours of that day at the beginning of the evening EMA. Furthermore, participants were asked about the activity they were currently involved in, providing information about possible work environment risk factors and/or information about their current work environment. We developed this activities scheme to be suitable for employees in academic settings and confirmed its structure and completeness with literature [26]. A flowchart of the activity-related questions can be found in Figure 5.

As encouragement, participants received a short feedback message included in the morning EMA on their response performance of the previous day they participated (e.g., on Monday they receive their participation results of the previous Friday).

##### EMA Triggering

The EMAs followed a schedule, personalized to participants’ wishes. The morning EMA was triggered randomly between 30 min and 60 min after the pre-set time of the start of work. In case a participant was not yet at their office at that time, they could postpone it for 30 min at a time. After the morning EMA, a daytime EMA was scheduled for every 90 min. This schedule only changed when a participant put off answering a questionnaire, so that triggering the next one would result in less than 30 min separation between two EMA sessions. The daytime EMAs continued until the participant indicated they had left their office. Finally, the evening EMA triggered at the pre-set time, before which the participant confirmed they had left the office or postponed it again for 30 min at a time. Edge cases of overtime work were also handled separately. This general schedule of EMAs repeated every weekday, but participants had the option of indicating they would not be working at their office on a particular day (e.g., in case of holidays) and the schedule continued the next working day as normal.

We wanted to minimize the number of missed notifications during the workday, so we took several steps to alert the users to them. We implemented additional notifications (re-reminders) for the daytime EMAs repeating every 90 min. If after the first notification the EMA was not answered in 15 min, a second notification was delivered. After this, no further notifications were issued for 75 min: while participants could still choose to answer the available questionnaire, they would not be disturbed again until the 90-min period was through. Participants also always had the option to snooze the notification: if they dismissed it (swiped it away), it reappeared after 15 min. Previous research has shown that such a snooze function generating reminder notifications increases participant adherence [12]. As an additional alert, if a notification was waiting, it appeared as a heads-up notification (in a floating window) on each smartphone unlock.

#### 2.2.2. Physiological Data

Physiological data were collected via the Empatica E4 wristband, which is an unobtrusive wrist device [27]. We felt that a wristband was the most convenient type of wearable device for monitoring physiological parameters. This particular device measures heart rate variability and electrodermal activity, being the most researched and most reliable parameters to detect stress, that can be provided via peripherally located physiological measurements. Further parameters measured by the Empatica E4 wristband were heart rate and skin temperature. Acceleration was measured to monitor context. The Empatica E4 wristband is one of few devices that combines all these features, and is probably the most mature and accurate, having been successfully used in many previous studies. We also felt that a wristband was the most convenient type of wearable device for monitoring physiological parameters. These two reasons led us to choose Empatica over a chest-worn ECG device, even though the latter would probably provide more accurate heart rate variability data.

Both the baseline measures of the physiological response during the first night and the two blood pressure measures (during the briefing and the debriefing) were used to predict blood pressure from the heart rate measures, which were collected via the Empatica wristbands, to improve stress prediction models.

An overview of all parameters measured and the assessment scheme can be found in Figure 2.

#### 2.2.3. Smartphone Sensor und Usage Data

Apart from serving as a platform for the EMAs, our STRAW app tracked smartphone sensor and usage data. Such data provided cues about contextual and environmental factors to improve stress prediction models, as demonstrated in previous research [28].

One sensor that was specifically developed and extensively tested for the STRAW project is the detection of human voice activity [29]. This sensor is relevant for our research question, since communication with others or more generally the social environment is a well-known work environment risk factor, as described in previous literature.

An overview of all parameters measured and the assessment scheme can be found in Figure 2. See Table A2 for a more detailed list and technical descriptions of them all, including the smartphone sensor and usage data.

### 2.3. Data Management and Privacy

The data from the EMA and smartphone sensor and usage data were stored on the smartphone and from there automatically transferred to a database. The data from the Empatica wristbands had to be transferred manually (approximately once per day) by the participants via the E4 Manager software to the E4 Connect cloud, which is a protected storage space provided by Empatica. The participants received their individual, automatically generated credentials to access the online survey via our STRAW website and to transfer their data from the Empatica wristband to the E4 Manager. During the data collection period, we were able to monitor incoming data and any potential technical issues via a self-developed dashboard and the E4 Connect cloud.

Within the STRAW project, private and sensitive data were collected. Several precautions were taken to protect the participants’ data with utmost care. User credentials and thereby access to the data were only given to the researchers directly involved in data management and analysis. All data were pseudonymized by replacing the participants’ full names with a participant ID, a random and automatically generated numeric identifier. The link between these two was stored separately from all other data. Additionally, location data were transformed so that the true location was never revealed, but the distances and recurring places can still be extracted.

The data were stored on the computer servers of the research institute in Slovenia, where servers dedicated to the STRAW project are accessible to authorized users only via the local internet connection or via a Virtual Private Network (VPN). An application vulnerability analysis carried out for the STRAW project showed that the risk of threats such as the data being hacked by an external party as very low.

We would like to emphasize that only communication metadata were stored. For example, the contents of messages, notifications, or audio data were never recorded or saved. The sole interest was the amount, timing, and length of notifications and human voice activity. Furthermore, the feedback report including their results was disseminated as a printed version via personal delivery of a researcher or the institutions’ internal mail service. These precautions taken to protect participants’ data according to the General Data Protection Regulation (GDPR) were emphasized in the informed consent form.

### 2.4. Stress Modelling and Data Analysis

We are going to approach stress modelling using different analytical strategies. We aim to improve our machine learning stress recognition models. While the existing models rely mainly on physiological data, this will be combined with participants’ behavior and context using the data from this study. These will be characterized both by features derived from smartphone usage (e.g., app usage) and non-digital activities (e.g., physical activity) tracked by the Empatica wristband. Smartphone-derived contexts will be considered potential causes and physiological data potential consequences of day-to-day stress. We will explore possibilities of combining all features into a single model and will incorporate contextual information (e.g., time of the day an EMA was responded to).

Our second aim is to investigate the relations between work environment risk factors and stress outcomes, while taking work activities and health-related behaviors into account. Traditional statistical methods will be used for this purpose, mainly multilevel statistical modelling for repeated data. The combination with physiological data and smartphone sensor and usage data will provide additional interesting results. This rich dataset will allow distinction between the effect of occupational stress due to objective work environment risk factors (e.g., work activities definable from the EMA and automated smartphone sensor and usage data) and due to subjective appraisal of occupational stress experienced [10].

## 3. Discussion

The STRAW project aims to disentangle and model relations between work environment risk factors, stress outcomes, physiological data, and context as inferred from smartphone sensor and usage data, within our target group of employees in academic settings.

This project provides a unique opportunity to advance stress research by integrating experience and expertise from two diverse fields: psychosocial occupational epidemiology and machine-learning disciplines. The results will be used to work further on personal health systems, where concepts of affective computing are becoming increasingly relevant to better understand users and achieve beneficial change in behavior. In addition to academic knowledge on occupational stress, the STRAW project has the potential for relevant practical impacts. Our results will provide concrete information on day-to-day stress experiences among academic employees, backed by solid evidence. This gives the possibility to advise on workplace procedures and policies, aiming to reduce stress at work in academic settings.

### 3.1. Strengths and Limitations

The main strength of the STRAW project is the combination of EMAs, physiological data, and smartphone sensor and usage data, which resulted in a rich dataset, providing the opportunity to explore occupational stress with subjective and objective measures, taking the work environment into account. The repeated measures allow research on within- and between-person levels and on relations between day-to-day fluctuations of work stress and chronic stress experiences. The EMA protocol contains a carefully planned and implemented content. Furthermore, the protocol by which the EMAs were triggered was customized not only to the STRAW project, but also to the individual participants. We developed the STRAW app over the course of over two years, built on our two pre-studies, previous research, and our pilot study, and we keep improving it throughout the data collection process. The STRAW app is backed by the self-developed dashboard to ensure that we receive and store all data safely, to maximize the quality and quantity of results we can get, and the conclusions we can draw. Due to the specificity of the target group and the work setting, transferability of the results might be possible to some extent to other sedentary office-based jobs, but generalizability to other occupational fields will be limited.

The main limitation of the STRAW project is the high demand on participants. Particularly, answering EMAs and wearing the Empatica wristband asks for time, effort, and adherence to the protocol from participants and ongoing involvement from the researchers during data collection to provide support. However, based on feedback from the pilot study participants, technical issues, worries about privacy, or other similar problems could be either solved or limited to an acceptable minimum. Furthermore, ongoing involvement from the researchers to offer support in case of any issues or uncertainties appeared to strengthen participant adherence. The main technical restriction was the limitation to Android smartphones, excluding iPhone users, which was due to restrictions on financial and human resources. A high amount of private and sensitive data were collected, primarily with the smartphone sensor and usage data. However, technical precautions were taken for all steps of the process, from tracking the data on the smartphones until final data analysis and result reporting.

### 3.2. Impact of the Covid-19 Pandemic

The currently ongoing Covid-19 situation influences our professional and personal lives and shapes the way of working of our target population, such as increased working from home or remotely in general. Due to this, we had to deal with long pauses in data collection.

In the initial protocol, participants were participating only on days when they were working at the office. We are now adapting this protocol and adjusting the data collection process so that participants can participate during working at home or remotely in general, as well. A further adaptation focuses on the work activities to the new way of working, such as adding “following an online activity (e.g., webinar, workshop, or course)” or adding the option of “teaching online” to the teaching and presenting options. Through this process we aim to adapt our research to novel ways of doing one’s job in the field of academia, being aware that the Covid-19 pandemic might have an influence on participants’ perception of occupational stress.

## 4. Conclusions

This study protocol describes the novelty of the STRAW project: combining an EMA study, physiological data, and smartphone sensor and usage data. The core of the project is the self-developed STRAW app, providing our participants of this study on day-to-day occupation stress with a carefully developed and well-designed data collection tool. Through the approach of collecting data in real-world work environments, we will be able to draw conclusions on which work environment risk factors cause day-to-day stress, which stress outcomes are experienced, in which work settings they occur, and how these results are interconnected. We aim to disentangle the phenomenon of occupational stress among academics, hoping to advance research and aiming to get results enabling us to provide practical advice to reduce stress at work and to prevent its adverse consequences.

## Appendix A

**Table A1:** Questionnaires included in the STRAW project.

Questionnaires	Online Survey	EMA
Pittsburgh Sleep Quality Index [30]	Included	Included
Job Content Questionnaire [7]	Included	Included
Work-Life Balance Inventory [31]	Included	Included
Perceptions of Fair Interpersonal Treatment Scale [32]	Included	Included
Utrecht Work Engagement Scale [33]	Included	Included
Recovery Experience Questionnaire [34]	Included	Included
COPE Inventory [35]	Included	Included
Effort Reward Imbalance Questionnaire [8]	Included	
Perceived Stress Scale [36]	Included	
Short Form - 12 [37]	Included	
Connor-Davidson Resilience Scale [38]	Included	
Positive and Negative Affect Schedule [39]		Included
Stress Appraisal Measure [40]		Included
Larsen and Kasimatis’ Symptoms Checklist [41]		Included

**Table A2:** List and technical descriptions of smartphone sensor and usage data.

Sensors	Description
Acceleration	There are several sources (i.e., virtual sensors) of acceleration data in a smartphone. Accelerometers measure acceleration magnitude in various directions and report either linear acceleration (without gravity effects), gravity, or combined acceleration. This is used further in Google’s activity recognition Application Programming Interface (API).
Applications	This includes the category of the application currently in use (i.e., running in the foreground) and data related to notifications that any application sends. Notification header text (but not content), the category of the application that triggered the notification and delivery modes (such as sound, vibration, and LED light) are logged.
Barometer	Ambient air pressure.
Battery	Battery information, such as current battery percentage level, voltage, and temperature, and its health, as well as power-related events, such as charging and discharging times are monitored.
Bluetooth	This sensor logs surrounding Bluetooth-enabled and visible devices, specifically their hashed Media Access Control (MAC) addresses, and Received Signal Strength Indicator (RSSI) in decibels.
Communication	Information about calls and messages sent or received by the user. This includes the call or message type (i.e., incoming, outgoing, or missed), length of the call session, and trace, a hashed phone number that was contacted. The phone numbers themselves or the contents of messages and calls were not logged.
Light	Luminance of the ambient light captured by the light sensor.
Location	Device’s current location (latitude, longitude, and altitude) and its velocity (speed and bearing). This uses various methods, such as GPS and known Wi-Fi’s in vicinity resulting in different degrees of accuracy. Location category is also acquired with Foursquare Application Programming Interface (API).
Network	Network availability (e.g., none or aeroplane mode, Wi-Fi, Bluetooth, GPS, or mobile) and traffic data (received and sent packets and bytes over either Wi-Fi or mobile data).
Processor	Processor load in Central Processing Unit (CPU) ticks and the percentage of load dedicated to user and system processes or idle load.
Proximity	Uses the sensor by the device’s display to detect nearby objects. It can either be a binary indicator of an object’s presence or the distance to the object.
Screen	Screen status: turned on or off and locked or unlocked.
Temperature	Temperature of the phone’s hardware sensor.
Time zone	Device’s current time zone.
Voice activity	A classifier trained using Weka. The features are calculated using openSMILE and the output is an indicator of human voice activity.
Wi-Fi	Logs of surrounding Wi-Fi access points, specifically their hashed Media Access Control (MAC) addresses, Received Signal Strength Indicator (RSSI) in decibels, security protocols, and band frequency. The information on the currently connected access point is also included.
